# Posterior Fossa Dysembryoplastic Neuroepithelial Tumor: A Neuropathological Report

**DOI:** 10.7759/cureus.33525

**Published:** 2023-01-09

**Authors:** George S Stoyanov, Lilyana Petkova, Toni Kondev, Radoslav Georgiev, Yavor Enchev

**Affiliations:** 1 General and Clinical Pathology, St. Marina University Hospital, Varna, BGR; 2 General and Clinical Pathology/Forensic Medicine and Deontology, Medical University of Varna, Varna, BGR; 3 Neurosurgery, Medical University of Varna, Varna, BGR; 4 Radiology and Radiotherapy, Medical University of Varna, Varna, BGR

**Keywords:** cerebellum, neurosurgery, pediatric tumor, neuropathology, dysembryoplastic neuroepithelial tumor

## Abstract

Dysembryoplastic neuroepithelial tumors (DNTs) are rare neoplastic entries of the central nervous system. Conventionally DNTs are with cortical location and predominantly occur in the temporal lobe associated with epilepsy. Subtentorial DNTs are rare entries. Herein we report a case of a two-year-old female with a DNT located in the cerebellum. The patient presented clinically with new onset gait instability, headaches and strabismus. Neuroradiology revealed a heterogenous, predominantly cystic lesion in the cerebellar vermis and left cerebellar hemisphere, which was interpreted as possible medulloblastoma based on the patient profile. Frozen section neuropathology was more suggestive of a low-grade glial tumor, with conventional histology and immunohistochemistry showing an admixture of glial and neuronal cells - a complex variety of DNT. Due to the histological appearance, differential diagnosis was required with other neuroglial tumors native to the posterior fossa, such as Lhermitte-Duclos disease. There have been several such published case reports, which, although of older patients, present with similar symptoms and neuropathological findings. The complexity of the neuropathological finding in posterior fossa DNTs can lead to future separation of this entry from conventional DNT, as was seen in the past with septum pellucidum DNT, now referred to as myxoid glioneuronal tumor.

## Introduction

Dysembryoplastic neuroepithelial tumor (DNT) is a rare central nervous system (CNS) tumor with a predominant cortical location, diagnosed almost exclusively in the pediatric population, with a male and Caucasian predominance [1-3]. The World Health Organization (WHO) defines DNT as a WHO grade 1 tumor, indicating its benign nature [4].

Clinically the main presenting feature is that of epileptic seizures, with around 6% of epilepsy cases being associated with DNT. The diagnostic criteria for the tumor include neuroimaging of a cortical mass without mass effect and peritumoral edema, sharply demarcated from the surrounding parenchyma, lobulated and a magnetic resonance imaging (MRI) fluid-attenuated inversion recovery (FLAIR) longitudinal relaxation time (T1) iso- or hypointensity and transverse relaxation time (T2) hyperintensity, with some tumor showing cystic and multicystic transformation [5]. Neuropathology criteria include the presence of glial columns oriented perpendicularly to the cortical surface, formed by bundles of axons lined by small oligodendroglia-like cells admixed with “floating” neurons [4,6]. Dysplastic ganglionic cells and abundant mitotic figures are absent. The glial component is highly heteromorphic with pyloid, oligodendroglioma-like and stellate features [7,8]. There are three subtypes of DNT: simple, complex, and diffuse/nonspecific; the simple form consists purely of the described glioneuronal elements; the complex further includes glial nodules, and the diffuse type while being controversial as it lacks the specific glioneuronal components, is comprised predominantly of oligodendroglioma-like cells [2,9].

DNTs are most often located in the temporal lobe, in nearly 70% of cases, with other CNS locations being rare [5]. So far, there have been only a handful of reports of DNTs with cerebellar locations [10-12].

## Case presentation

**Figure 1:**
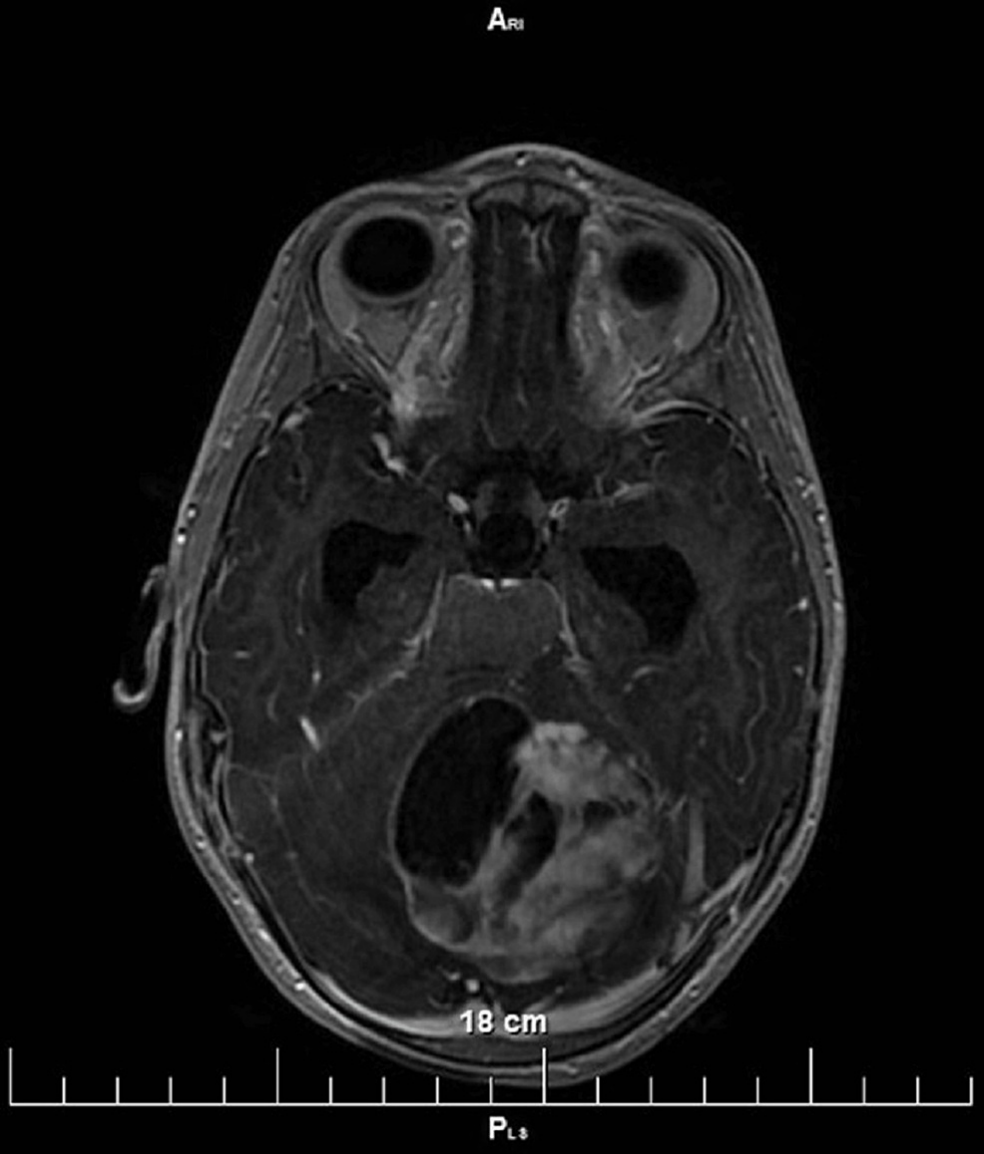
AX 3DT1 native MRI of the brain showing a cystic tumor formation in the left cerebellar hemisphere AX 3DT1: axial three-dimensional longitudinal relaxation time; MRI: magnetic resonance imaging

The patient, a two-year-old female, presented to our institution with new onset gait instability, headaches, and strabismus steadily developing over the past two months. Pregnancy, delivery, neonatal, and early developmental period were uneventful, with no significant medication use. Pediatric neurology revealed only gait instability without any additional findings. A CNS MRI revealed a posterior fossa multicystic tumor involving the cerebellar vermis and left cerebellar hemisphere, with a T1 hypointensity and T2 hyperintensity, without significant peritumoral edema and a largest diameter of 48mm, compressing the fourth ventricle and leading to dilation of the ventricular system (internal hydrocephalus) (Figure 1). The tumor was interpreted as a possible medulloblastoma based on the patient's profile and the clinical and neuroradiological findings.

**Figure 2:**
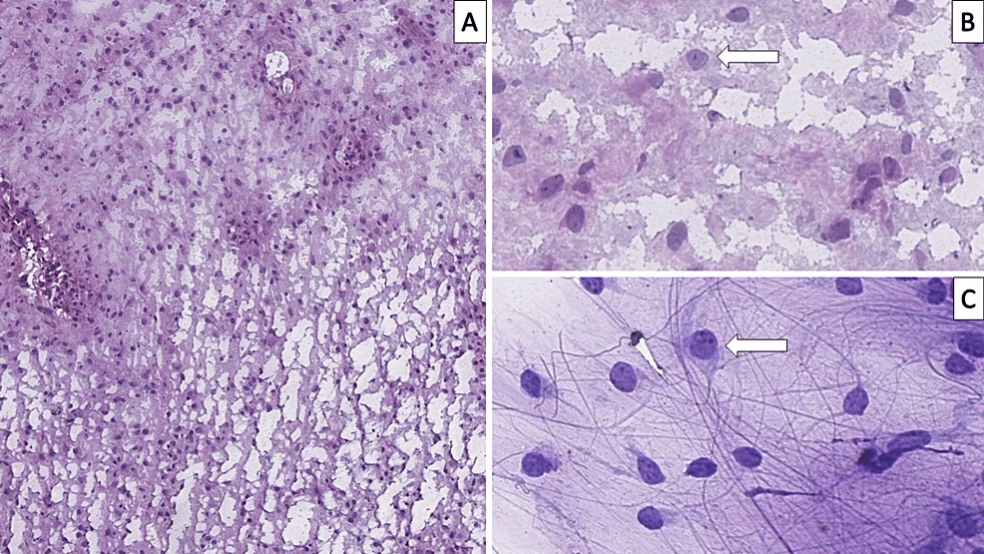
Frozen section neuropathology of the tumor A: cellularity suggestive of a low-grade tumor with cystic areas, original magnification 100x; higher magnification from plain A suggestive of a neuronal component (arrow), original magnification 400x; C: cytology showing abundant neuropil, tumor cell with scant cytoplasm and a cell with neuronal morphology (arrow), original magnification 400x

Based on the neuroradiological finding, the patient was scheduled for immediate neurosurgical intervention. During surgery, the tumor was noted to be myxoid and easily bleeding. A specimen was sent for frozen section neuropathology and interpreted as a low-grade glial neoplasm (Figures 2A-2C). Due to the sharp margin with the surrounding tissue, gross total excision was achieved. The postoperative period was uneventful, and the patient remained in stable condition.

**Figure 3:**
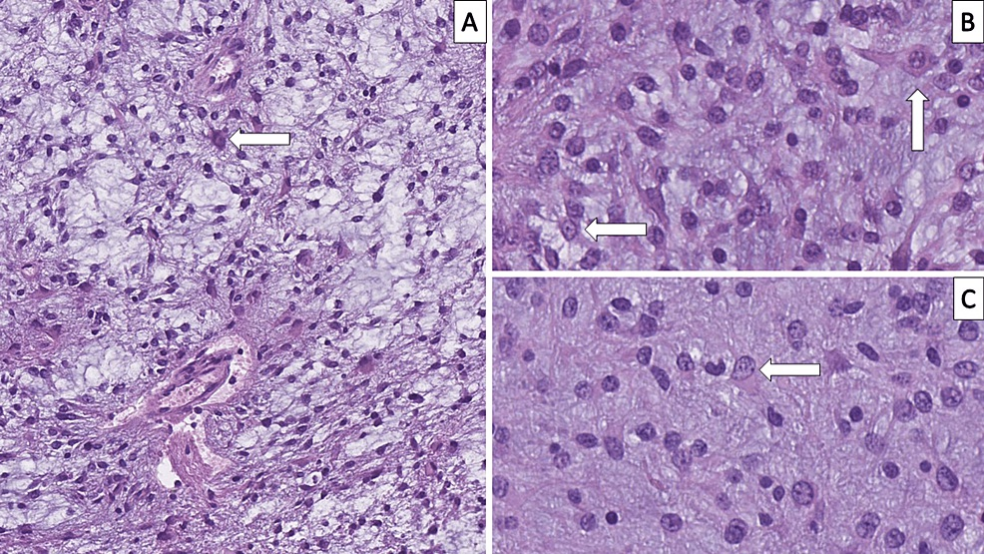
Histopathology of the tumor (A) Malformative blood vessels, myxoid areas, cords of tumor cells, some with oligodendroglioma-like morphology and floating neurons (arrow), hematoxylin and eosin stain, original magnification 200x; (B) floating neurons (arrows), hematoxylin and eosin stain, original magnification 400x; (C) solid astrocytoma-like areas of the tumor with floating neurons (arrow), hematoxylin and eosin stain, original magnification 400x

Formalin-fixed paraffin-embedded tissue sections (FFPETS) from the tumor revealed parts of the cerebellar cortex with a tumor proliferation involving both the cortex and white matter of the cerebellum comprising of a myxoid stroma with admixed glioneuronal components with small oligodendroglioma-like cells, pyloid astrocytes, and floating neurons. Areas of microcystic degeneration, myxoidisation, and malformative blood vessels were also noted throughout the specimen (Figures 3A-3C).

**Figure 4:**
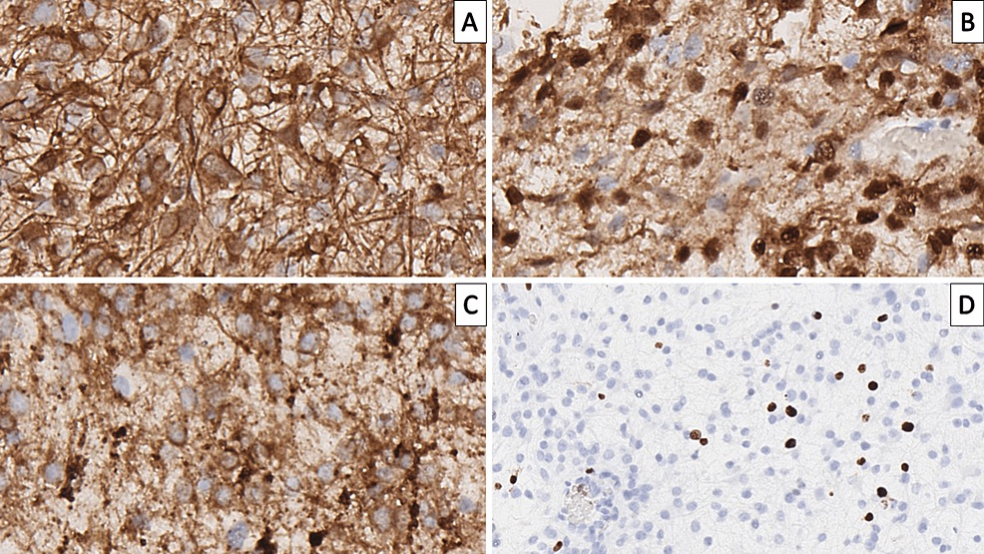
Immunophenotype of the tumor (A) Glial fibrillary acidic protein underlining the glial component of the tumor, original magnification 400x; (B) S100 protein, original magnification 400x; (C) Synaptophysin, original magnification 400x; (D) high Ki-67 proliferative index; original magnification 200x

**Figure 5:**
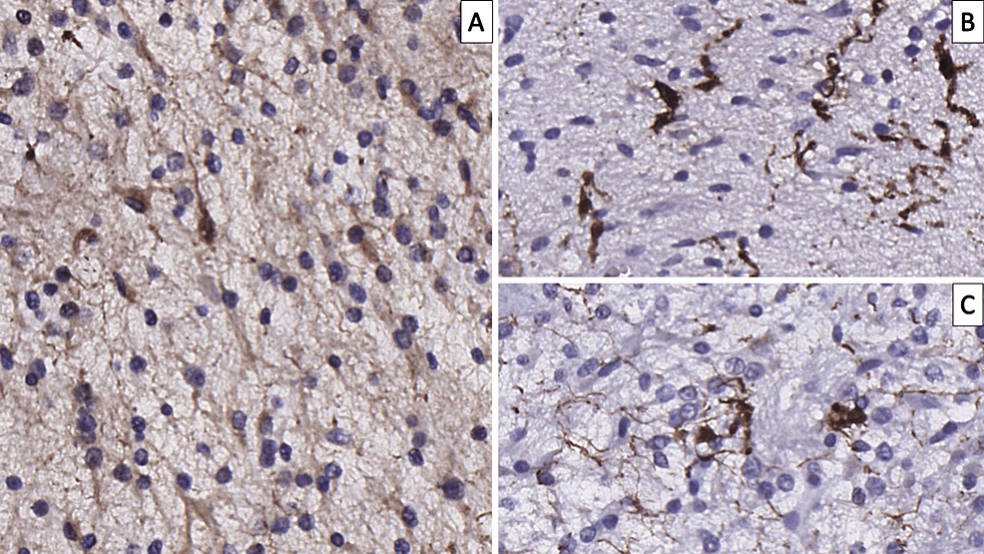
Immunophenotype of the tumor regarding the neuronal component (A) Neuron specific enolase, original magnification 400x; (B, C) neurofilament, original magnifications 400x

Immunohistochemistry revealed S100 protein expression in the oligodendroglioma-like cellular component, glial fibrillary acidic protein (GFAP) in the astrocytic components, synaptophysin positivity neuron-specific enolase and neurofilament expression in the floating neurons, Ki-67 proliferative index was high in regard to tumor cellularity (Figures 4A-4D, 5A-5C). Based on the morphological finding and the immunohistochemical profile of the cellular components, the diagnosis of complex cerebellar DNT, WHO CNS grade 1, was defined.

**Figure 6:**
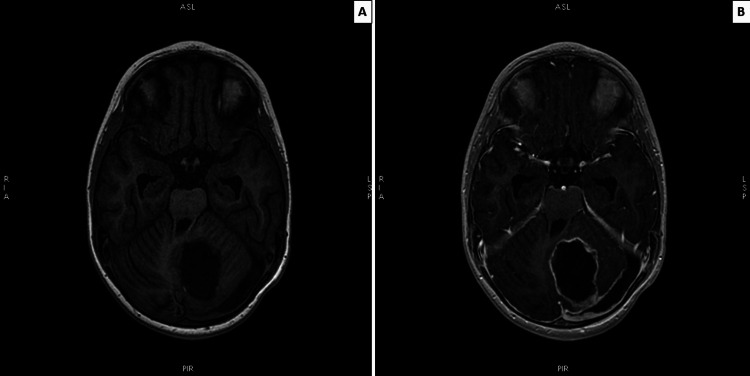
(A) AX 3DT1 native and (B) AX 3DT1 postcontrast MRI of the brain showing postoperative changes in the left cerebellar hemisphere-porencephalyc cyst with peripheral gliosis and hemosiderin deposits, with peripheral contrast enhancement AX 3DT1: axial three-dimensional longitudinal relaxation time; MRI: magnetic resonance imaging

One and a half years after the operation, the patient is stable, with improved neurological status and good neuropsychiatric development. Regular neuroradiology shows no recurrence of the disease and resolution of the hydrocephalus (Figures 6A, 6B).

## Discussion

The principal differential diagnosis in our case is neoplasms native to the posterior cranial fossa, mainly medulloblastoma [4]. Within the context of our case, despite medulloblastoma being the most common cerebellar tumor in this age group, neuroradiology did not show invasive growth and, although suggestive, did not completely confirm the neuroradiological findings as such. Furthermore, frozen section neuropathology, as seen in Figure 2, depicted a significantly less cellular neoplasm than medulloblastoma, which was confirmed on FFPETS. The presence of a neuronal component and the admixture of varying glial cell fractions necessitated the differential diagnosis between neuroglial neoplasms, one of which dysplastic cerebellar gangliocytoma, also known as Lhermitte-Duclos disease, is native to the cerebellum, as well as others such as ganglioglioma, myxoid glioneuronal tumor (MGT), multinodular and vacuolating neuronal tumor and gangliocytoma [4,13-15]. While there are multiple other entries in the glioneuronal and neuronal tumors, the architecture and cellularity contradict their diagnosis in our case. Furthermore, despite there being a prevalent neuronal component in our case, the neurons present were not ganglion-type neurons, excluding all of the aforementioned tumor entries, except MGT.

As per the 2021 WHO classification of nervous system tumors, MGT is a newly defined entry, previously identified as a DNT-like neoplasm, most commonly diagnosed in the pediatric population and with an identical neuroradiological finding; however, despite classically being located in periventricular areas, most often it is not associated with the cortex, but with the septum pellucidum [4,14].

Furthermore, in our case, as seen in Figure 3, there are some non-classical features of DNT, such as blood vessels horizontal to the cerebellar cortex and cytoplasmic projections of the glial component towards those blood vessels. The presence of non-classical components such as the depicted ones and areas of more compact growth, without conventional morphology reminiscent of other glial tumors, such as astrocytoma and oligodendroglioma further expand the diagnostic spectrum and necessitates such cases to be diagnosed as complex varieties of DNT [15].

To date, there have been only a few reported cases of DNT with cerebellar location, with Yuan et al. reporting a case that is virtually identical to ours, both in the patient profile and the presenting symptoms [12]. Other similar cases have also been published, such as those of Fujimoto et al., Kuchelmeister et al., and Sunwoo and Kim; however, the patients presented are all adults, unlike in our case [16-18].

Based on the previous separation in the classification of DNT and DNT-like tumors of the septum pellucidum, it is likely that based on the complex morphology of cerebellar DNT tumors, that they would also be separated from this group in the future as a unique entry.

## Conclusions

DNT is a rare glioneuronal tumor classically located in the temporal lobe and associated with the cerebral cortex, clinically manifesting with epileptic seizures in both the pediatric and adult populations. The presented case enriches the spectrum of DNT with cerebellar locations, which as exceedingly rare entries, necessitate a broad set of differential diagnoses both on their clinical manifestation, and neuroradiological and neuropathological findings.

Unlike DNTs with a classical location and furthermore, in a pediatric patient, our case presented with new onset gait instability and strabismus, which, based on the few similar cases published so far, can be considered a classical feature of the tumor in this location.
